# Improving sensitivity of amyloid detection by Congo red stain by using polarizing microscope and avoiding pitfalls

**DOI:** 10.1186/s13000-019-0822-4

**Published:** 2019-06-14

**Authors:** Ashraf El-Meanawy, Christopher Mueller, Kenneth A. Iczkowski

**Affiliations:** 1 Department of Medicine, Medical College of Wisconsin, 8701 Watertown Plank Rd, Milwaukee, WI 53226 USA; 2 Departments of Pathology, Medical College of Wisconsin, Milwaukee, WI USA

**Keywords:** Amyloidosis, Metallurgical microscope, Polarized microscopy, Congo red

## Abstract

Systemic amyloidosis is a devastating group of disorders for which there is no current cure. The treatment goal is to reduce the burden of amyloidogenic protein precursors. The treatment is only effective if applied early in the disease process before significant and irreversible end organ damage has taken place. Congo red is still the standard stain used in most histopathology laboratories to identify amyloid material in tissues. The identification of Congophilic amyloid material is challenging because of multiple interfering factors. Here we describe improved sensitivity of identifying Congophilic materials in histologic sections using a metallurgical polarized microscope specifically constructed for polarized microscopy. The microscope is equipped with strain-free optics, matching polarizers, dis-integrated compensators, and a circular mobile stage. Compared to a standard clinical microscope, this setup significantly improves sensitivity of identifying amyloid material in Congo red-stained slides. We also describe the deleterious effect of plastic coverslip which can interfere with the ability to examine the slides under polarized light. We present a series of 10 different patients who had cardiac, brain, and salivary gland biopsies that were either equivocal or deemed negative using a standard clinical microscope but were positive using the equipment described above. These samples were confirmed to be positive by other methods including electron microscopy. We conclude that use of the correct equipment is needed before ruling out amyloidosis in tissue sections.

**Electronic supplementary material:**

The online version of this article (10.1186/s13000-019-0822-4) contains supplementary material, which is available to authorized users.

## Background

Amyloidosis is a group of disorders caused by protein misfolding and aggregation. Systemic amyloidosis is a consequence of circulating amyloidogenic protein monomers which deposit in various tissues with variable affinities, causing tissue damage and multi-organ dysfunction. The term amyloid is a misnomer based on Rudolf Virchow’s mistakenly identifying the material as starch (Amylin) [1]. More than 40 different proteins have been identified as a precursor for amyloid formation in humans [2]. The most common forms of systemic amyloid are AL amyloid seen in plasma cell dyscrasias, AA amyloid associated with inflammatory conditions, and TTR amyloidosis due to either familial gene mutation or wild type protein, formerly called senile amyloidosis. The clinical picture and prognosis of amyloidosis is dependent on organ(s) affected and whether organ dysfunction can be identified by symptoms or quantified by functional testing, particularly heart, kidney, and nervous system which are frequently involved and trigger testing.

Cardiac involvement is manifest in approximately one-third to one-half of all AL patients at the time of diagnosis [3]. The predominant presenting symptom is rapidly progressive heart failure with preserved ejection fraction. Unfortunately, symptoms of cardiac decompensation are also a major risk factor for mortality. Kyle et al. showed that in 168 patients with systemic amyloidosis, those who presented with congestive heart failure had a median survival of 4 months [4]. Renal amyloidosis is frequently, but not necessarily always, associated with high grade proteinuria. The trigger for amyloid workup is usually nephrotic syndrome and/or decline of renal function. Renal amyloidosis is not as rare as it was once thought to be. Retrospective analysis of 17 years of renal biopsies in the Czech Republic revealed that 43% of cases of nephrotic syndrome above the age of 60 were due to amyloidosis [5]. Neuropathy is another significant manifestation of neuron-avid monomers, which can be devastating leading to peripheral and/or autonomic neuropathies with crippling manifestations [6, 7].

The frequently observed rapid functional recovery of kidney and heart with therapies that limit or abolish monomer production is often attributed to direct monomer and/or oligomer toxicity [8, 9]. But, it also underscores the imperative of establishing the diagnosis of amyloid as early as possible so that effective therapy can be instituted. Unfortunately, the diagnosis of amyloid in tissue sections is challenging. Identification of fibrils by electron microscopy (EM) is highly specific but because of the patchy nature of the disease, and the magnification level of EM, the sensitivity is very poor a region of interest can be identified using another method. Thioflavin-T is very sensitive [10, 11] but its specificity is not trusted by some experts. Interpretation of Thioflavin-T staining is also plagued by its tendency to bleach, as well as the subjective assessment of intensity based on comparison to a very strong positive control. Congo red, despite having lower sensitivity, is the standard agent used to identify amyloid in tissues. While the apple-green birefringence seen under crossed polarized light is specific for amyloid material, staining with Congo red is technically difficult resulting in inconsistent staining. Moreover, variation of mounting media and limitations of the examining microscopes increase both false-negative and false-positive results. Pitfalls of the staining techniques have been addressed elsewhere [12–14]. In this paper we show that the use of a microscope built specifically for polarized light increases sensitivity of identifying amyloid in Congo red stained sections. We also describe minor pitfalls in examination of Congo red stained slides.

## Methods

The metallurgical microscope used in the current report is built specifically for polarized microscopy. These microscopes are widely available from different manufactures and cost between $13,000–$20,000 which is comparable to standard clinical microscopes. The microscope is equipped with strain free objectives, condenser, and eyepieces. The strain free optics is critical to eliminate the false optical effects generated by stressed glass under polarized light. These spurious artifacts can interfere with the ability to evaluate birefringence produced by Congo red stained amyloid deposits. The condenser, besides having strain free lenses, is designed to produce perfect parallel beam of light. One of the most important features of a polarized microscope is the 360° circular rotating stage. This allow easy and full rotation of each examined field in the specimen. The microscopes we used have a fixed polarizer fitted in the condenser and an analyzer with a precise degree dial for cross setup. The illustration accompanying the test is generic and can apply to any polarized microscope (Microscope illustration).

Such microscope is ideal for polarized microscopy and is superior to a clinical microscope for examining Congo red stained sections. We obtained similar results using similar microscopes from Leica and Nikon. Using Sénarmont compensator did not provide added benefit. The same slides were also examined using a clinical microscope in which the analyzer has a built-in λ compensator, which is common equipment sold with clinical microscopes. The λ compensators are useful in examining crystals such as uric acid but can hamper detection of amyloid deposits. All the polarized microscopes were equipped with a circular rotating mechanical stage.

We specifically selected cardiac, salivary gland, and brain biopsies, which were initially deemed negative when examined by clinical microscope but were later confirmed positive for amyloidosis. These cases cover 3 types of amyloid AL, ATTR, and FGA (Table 1). Regardless of the amyloid type, the polarized microscope was superior to clinical microscope. Commercially purchased positive controls were also examined to demonstrate the validity of the techniques. Although Congo staining technique vary from lab to lab, we found no significant difference between in-house prepared stain, vs Leica, vs Dayko stains

**Table 1:**
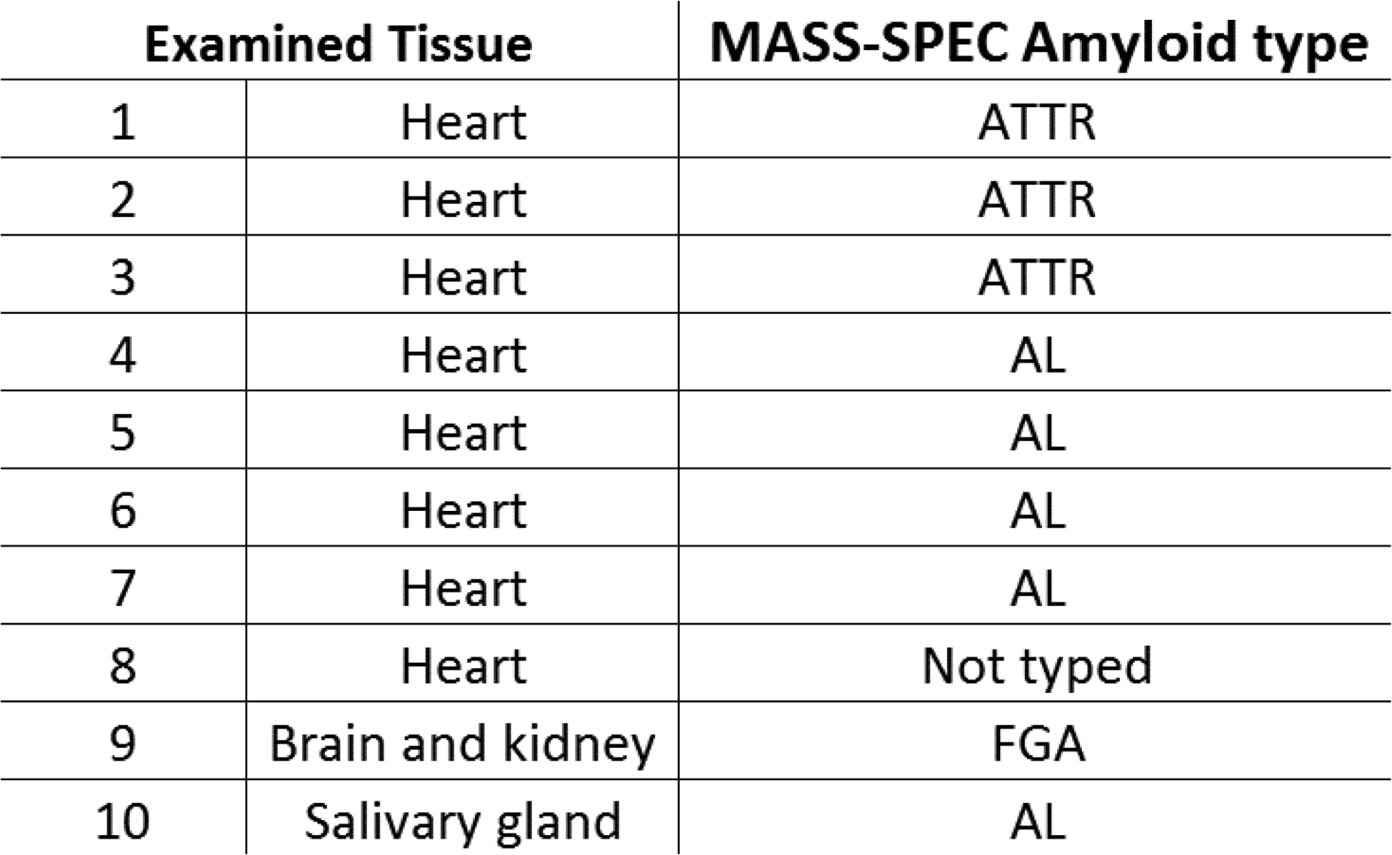
List of reviewed cases, tissue examined, and amyloid type

## Results

**Fig. 1:**
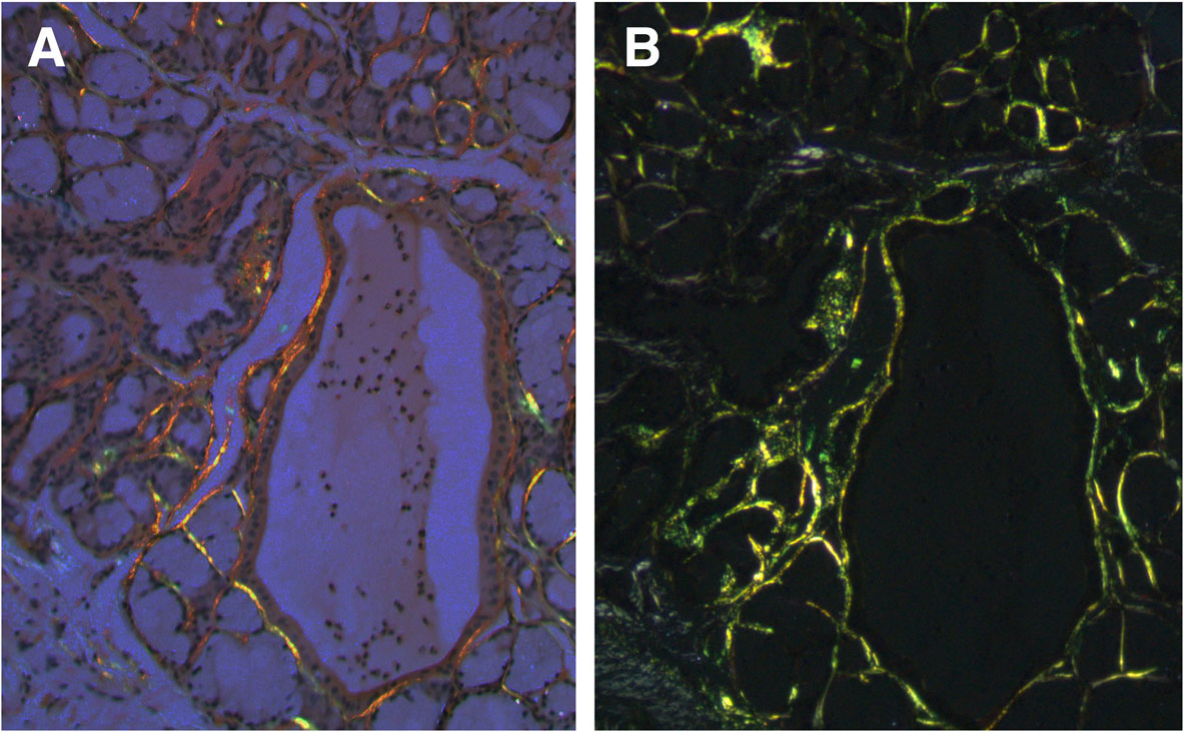
Congo red stained salivary gland section examined under crossed polarized light from patient with AL amyloid imaged using clinical microscope (A) and same field examined using Metallurgical microscope

**Fig. 2:**
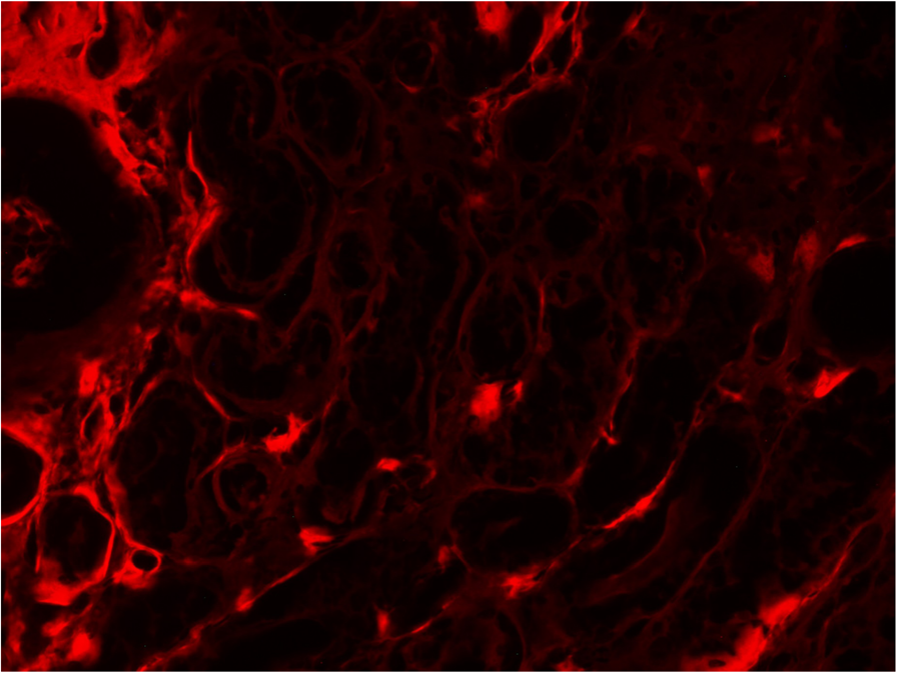
Congo red examined with fluorescent microscope using a Texas-red filter

We found that the apple green birefringence is more readily visible and with higher intensity when the slides are examined using a metallurgical microscope compared to the standard clinical microscope. Fig. 1 shows an image of biopsies examined by the metallurgical microscope for comparison purpose, the representative same field imaged with a standard microscope is shown. Additional file 1 has a series of biopsy samples examined by clinical and metallurgical microscope. As it is known that Congo red can be fluorescent, we examined the slides with fluorescent microscope using Texas red filter. Figure 2 shows representative image displaying the red fluorescence of Congo red in tissues. Fluorescence is more sensitive but less specific for amyloid stained with Congo red [15].

### A plastic coverslip can obliterate the ability to examine slides properly

**Fig. 3:**
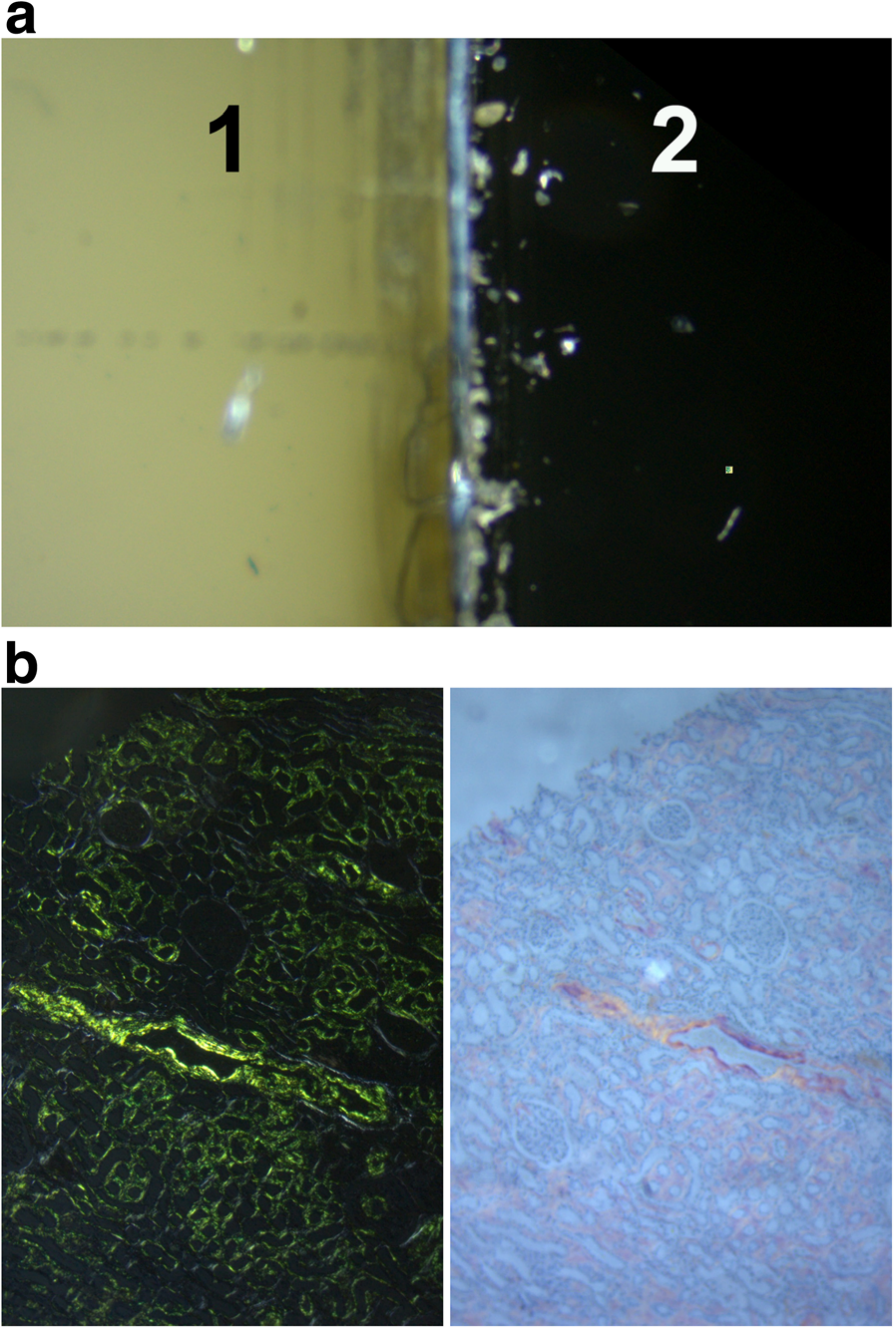
**a** Negative impact of plastic coverslips in polarized microscopy, due to disorganized polarizing effect of some plastic polymers, it is difficult to obtain proper crossing of polarized light and light pass through on cover slip side (1) and proper dark field without the coverslip (2). **b** Negative impact of plastic cover slip in polarized microscopy showing positive control sample examined with glass cover slip (left) or plastic coverslip (right)

We examined 2 samples (kidney and heart) from 2 different institutions where plastic cover slips were used. We found that plastic coverslips scatter light and inconsistently polarize in a pattern that makes it impossible to cross the microscope polarizer and analyzer properly to obtain a dark field. Figure 3a shows a comparison of the light passed through a slide with plastic cover slip at the edge of the cover. The plastic coverslip allowed the light to go through while glass did not. This prevents examining the slide under crossed polarized light. Unfortunately, the outside labs exhausted both biopsy tissues from these patients and it was not possible to procure more material. To further examine the effect of plastic coverslips we obtain cover slips from 2 different manufacturers. Both have ill effect on examining Congo red stained sections. Figure 3b showed positive control viewed through glass and plastic coverslips. We also observed that plastic effect was different depending on the manufacturer and as shown when rotating the slides. Video recording of rotated slides covered with glass or plastic coverslips is seen in Additional file 1.

### Rotating slides using a circular stage is important

**Fig. 4:**
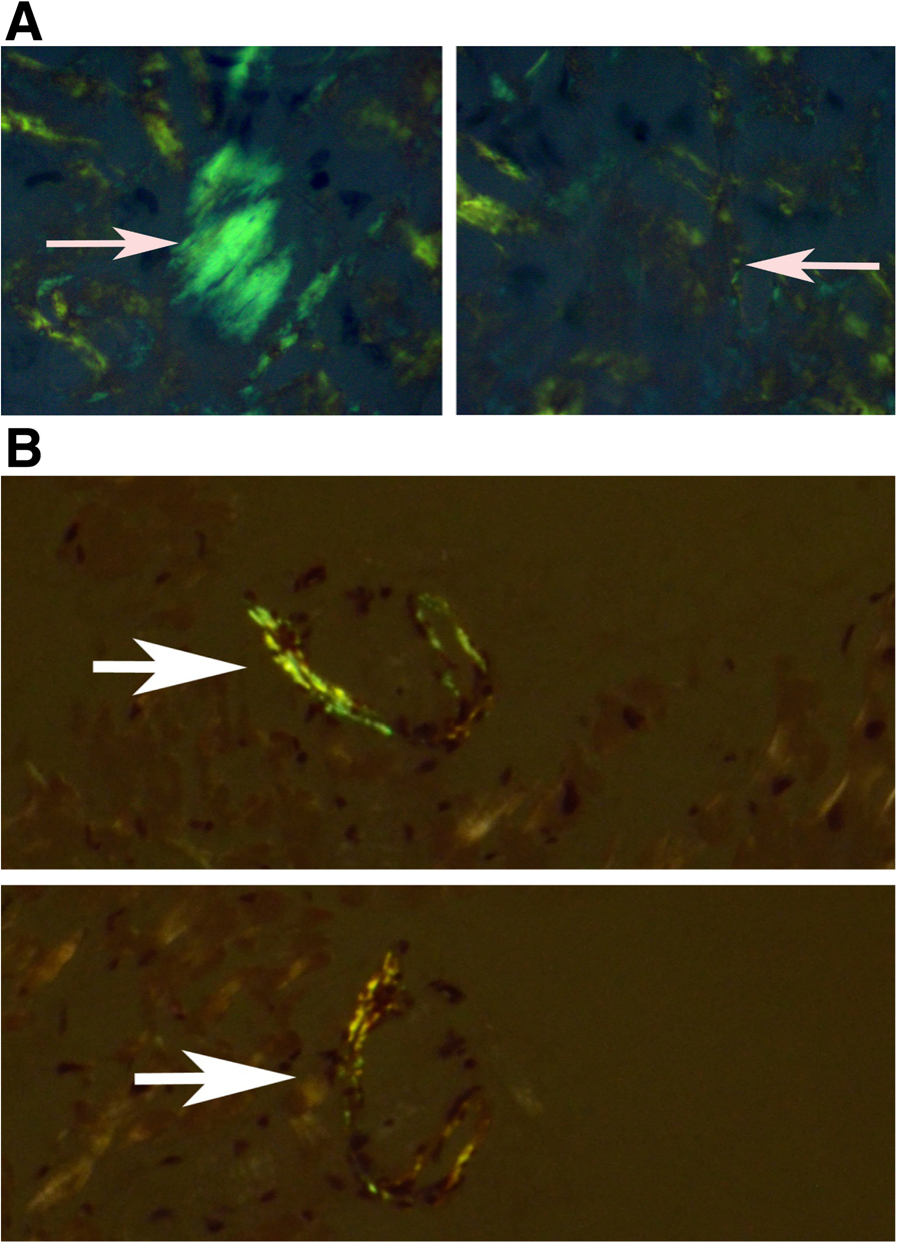
shows a sample imaged then re-images after rotating the stage. 4A rotated 45 degrees and 4b rotated 60 degrees. The green birefringence is no longer visible as indicated by the arrows

The orientation of the Congo red stained amyloid fibrils in relation to the plane of the light path can alter detection. Figure 4 shows samples imaged, then re-imaged after rotating the stage. It is evident that the apple green birefringence can be seen only at one angle. This highlights the need to use a mechanical circular stage, not found on clinical microscopes.

### Blue hue

**Fig. 5:**
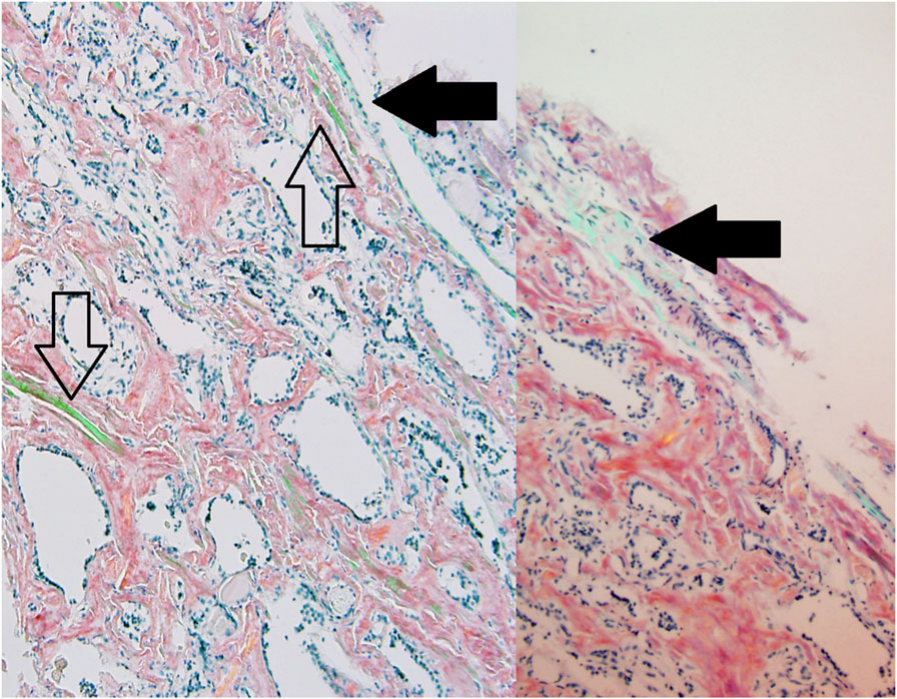
Congo red stained slide images using standard microscope with an analyzer that has a build-in compensator. Blue hue (black arrows) compared to apple green (frame arrows). Sometimes this is caused by partial uncrossing of an analyzer with built in compensator

A common clinical analyzer has a polarizing filter with a built-in compensator which usually adds color and is often used for crystal examination. The use of such an analyzer in examining Congo red stained specimens, frequently leads to a field that is excessively bright and sometimes forces the observer to partially uncross the analyzer in order to accentuate the apple green birefringence. However, this results in appearance or enhancement of a bluish-green hue (Fig. 5) mainly due to collagen and other matrix proteins [16, 17].

## Discussion

In this report, we show that suitable microscopy equipment can increase the sensitivity of identifying the amyloid-specific birefringence in Congo red-stained tissue sections. Early diagnosis of systemic amyloidosis is essential to reducing morbidity and mortality of the disease. Despite the seriousness of the disease and the benefit of early detection, an accurate pathologic diagnosis is still challenging. Spotty nature of disease, variation in organ-to-organ of the density of amyloid deposits, and the difficult of reproducible tissue staining, all increase the odds of false negative and false positive results. A negative tissue pathology report can effectively exclude the diagnosis of amyloidosis, which is then frequently never reconsidered among differential.

The standard of care for multiple myeloma, mono, and polyclonal gammopathy is conservative follow up, unless there is identifiable end-organ damage or amyloidosis [18]. Accordingly, missing an early diagnosis of amyloidosis can deprive a patient from receiving lifesaving treatment and can lead to costly and sometimes invasive investigations to pursue alternative diagnoses. In the case of transthyretin or fibrinogen amyloidosis, early liver transplantation usually arrests disease progression and can even be curative [19–21]. Therefore, even a marginal improvement in sensitivity of detection of amyloid in tissue specimens will help in assuring that patients with this serious and frequently fatal disease can be treated promptly and receive accurate prognostic information.

The real prevalence of amyloidosis is not known. A retrospective evaluation of kidney biopsies suggests that amyloidosis is not as rare as it is thought to be accounting to 43% of nephrotic proteinuria above age of 60 [5].

In single center experience 31% of patients with multiple myeloma patients has had confirmed evidence of amyloidosis [22]. The prevalence of multiple myeloma is dwarfed by the prevalence of monoclonal gammopathy, which can be as high as 8.4% depending on the race [23–25].

When the diagnosis is missed discipline-specific bias leads to ascribing to organ dysfunction to diabetes in case of renal disease and neuropathy and cardiac symptoms on hypertension or ischemia. Yet, there is no systematic data that examine the accuracy of these presumptive etiologies, and it is not inconceivable that some fraction of these patients may be incorrectly classified.

Owing to the patchy nature of amyloidosis, especially during its early stages, amyloid deposition could be restricted to just a small area of the tissue biopsy only visible at a limited angle of slide viewing. Thorough examination of each section using a mechanical rotating stage to view slides at variable angles is essential to avoid missing such deposits. We also recommend that plastic cover slips be avoided as they can interfere with the ability to perform crossed polarized light examination and reduce ability to identify subtle or low density amyloid deposits. Low density deposits are enough to make the diagnosis due to the patchy nature of the disease. When a sample is deemed negative or equivocal, there is a need to follow previous published modifications like the use of polar mounting media or omitting the alcohol differentiation step when examining collagen rich tissue to avoid interference [26–29]. Finally, the use of proper optics like those of a metallurgical microscope is essential to avoid missing the presence of small deposits of amyloid in Congo red-stained tissue.

## Conclusions

There is variability on the reporting of Congo red stained slides between different labs and pathologists. We identified important pearls that can improve the ability to identify amyloid material in Congo red stained tissues. We found that it is critical to use microscope with proper strain free optics and avoid the use of polarizer with built-in compensator. The use of mechanical rotating stage will reduce the chance of missing subtle or low-level amyloid deposits which can only produce birefringence at specific angles. Last, plastic cover slips can lead to inability to examine the slides under crossed polarized light. Improving sensitivity of the Congo red evaluation can aid in early diagnosis of amyloid and will have tremendous impact on clinical outcome of some patients.

## Additional file

Additional file 1: Included a video recording of congored stained tissue covered by eith glass or plastic coverslips and rotated. (PPTX 11971 kb)

## Abbreviation

AA: Amyloid-A
AL: Amyloid Light chain
EM: Electron microscopy
TTR: Transthyretin
