# Supplementary material for: Improving sensitivity of amyloid detection by Congo red stain by using polarizing microscope and avoiding pitfalls
Source: Diagn Pathol. 2019 Jun 14;14:57. doi: 10.1186/s13000-019-0822-4 (PMC6567537; doi:10.1186/s13000-019-0822-4)

## Slide 1
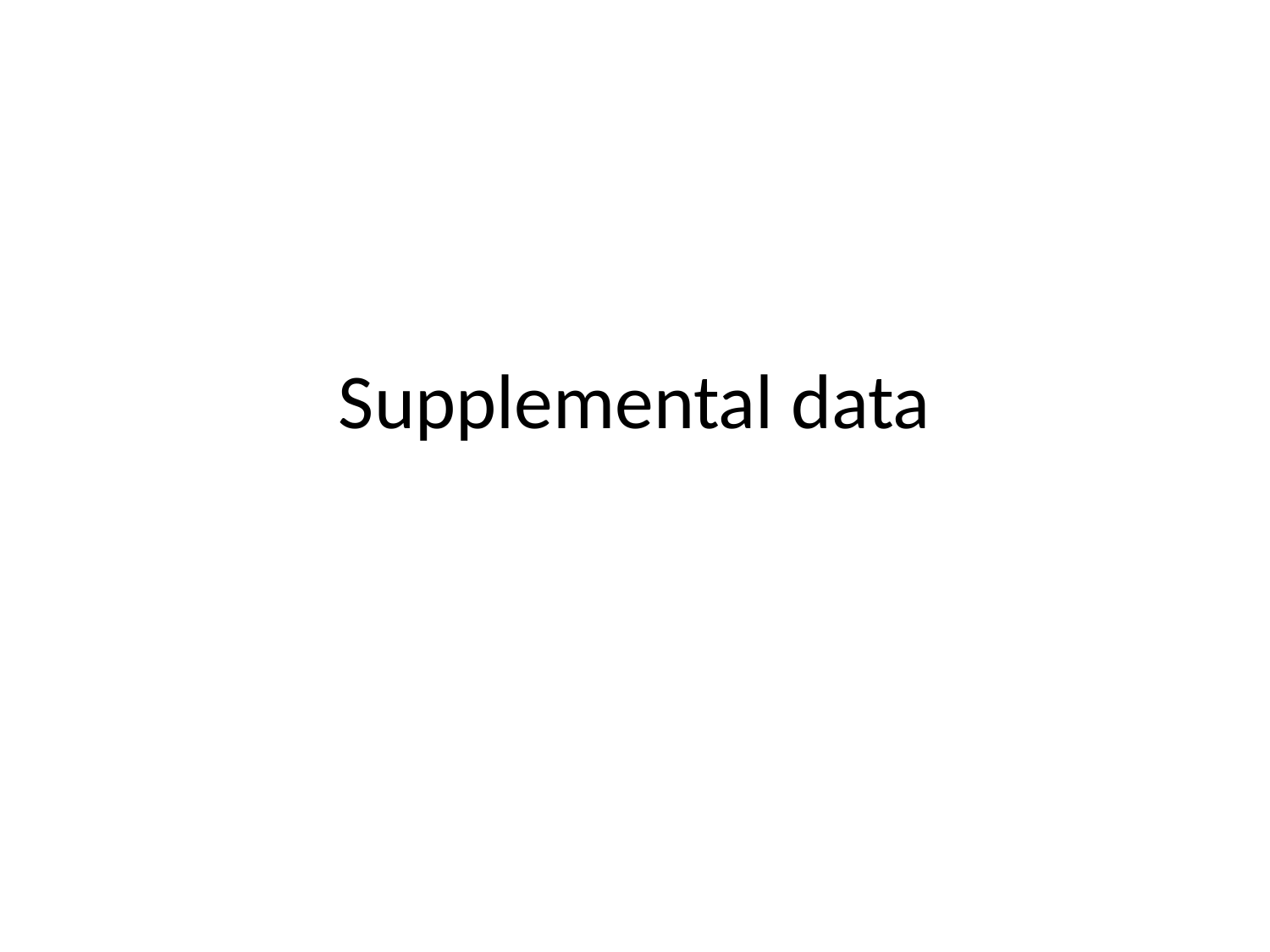

# Supplemental data

## Slide 2
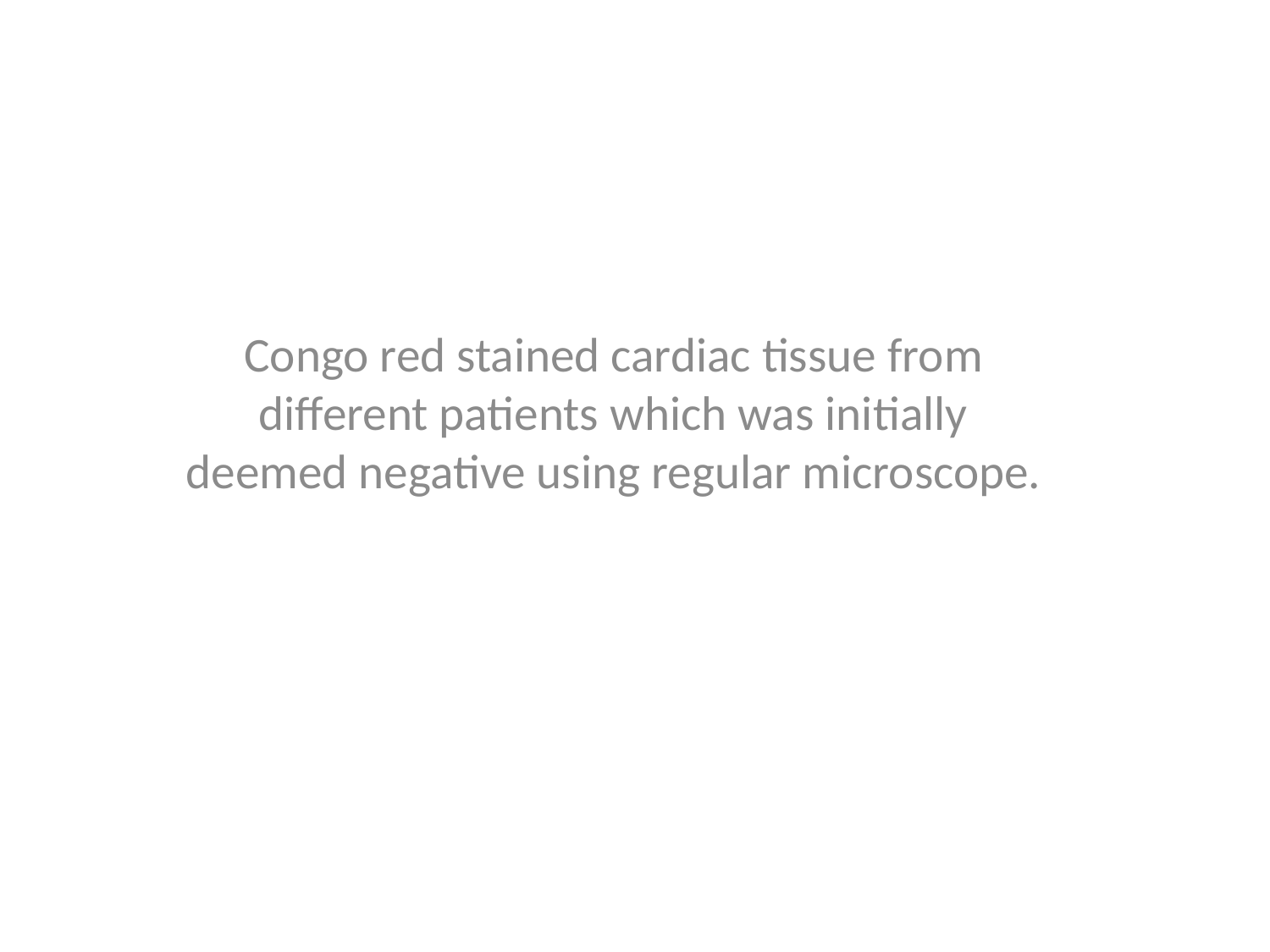

Congo red stained cardiac tissue from different patients which was initially deemed negative using regular microscope.

## Slide 3
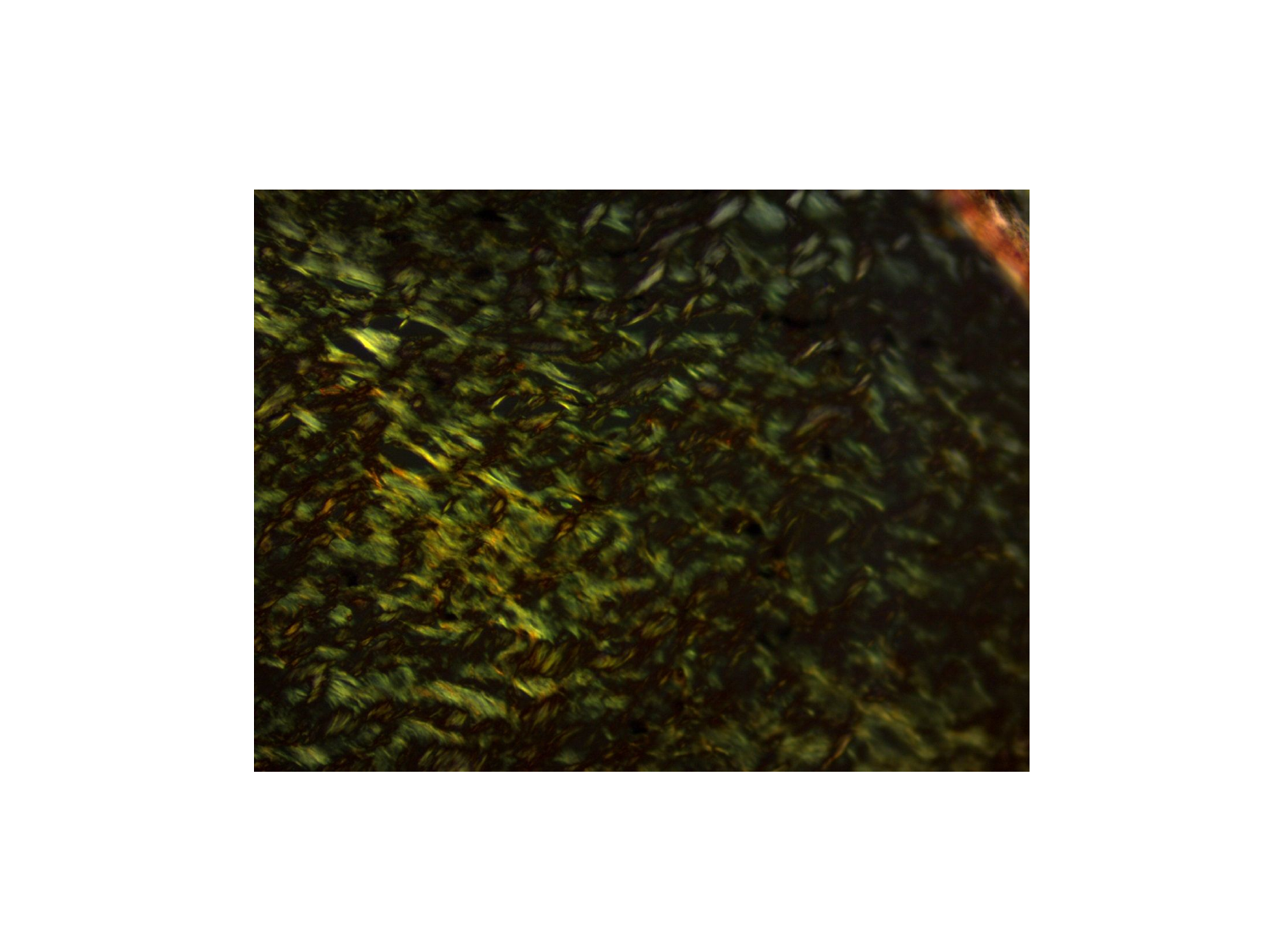

## Slide 4
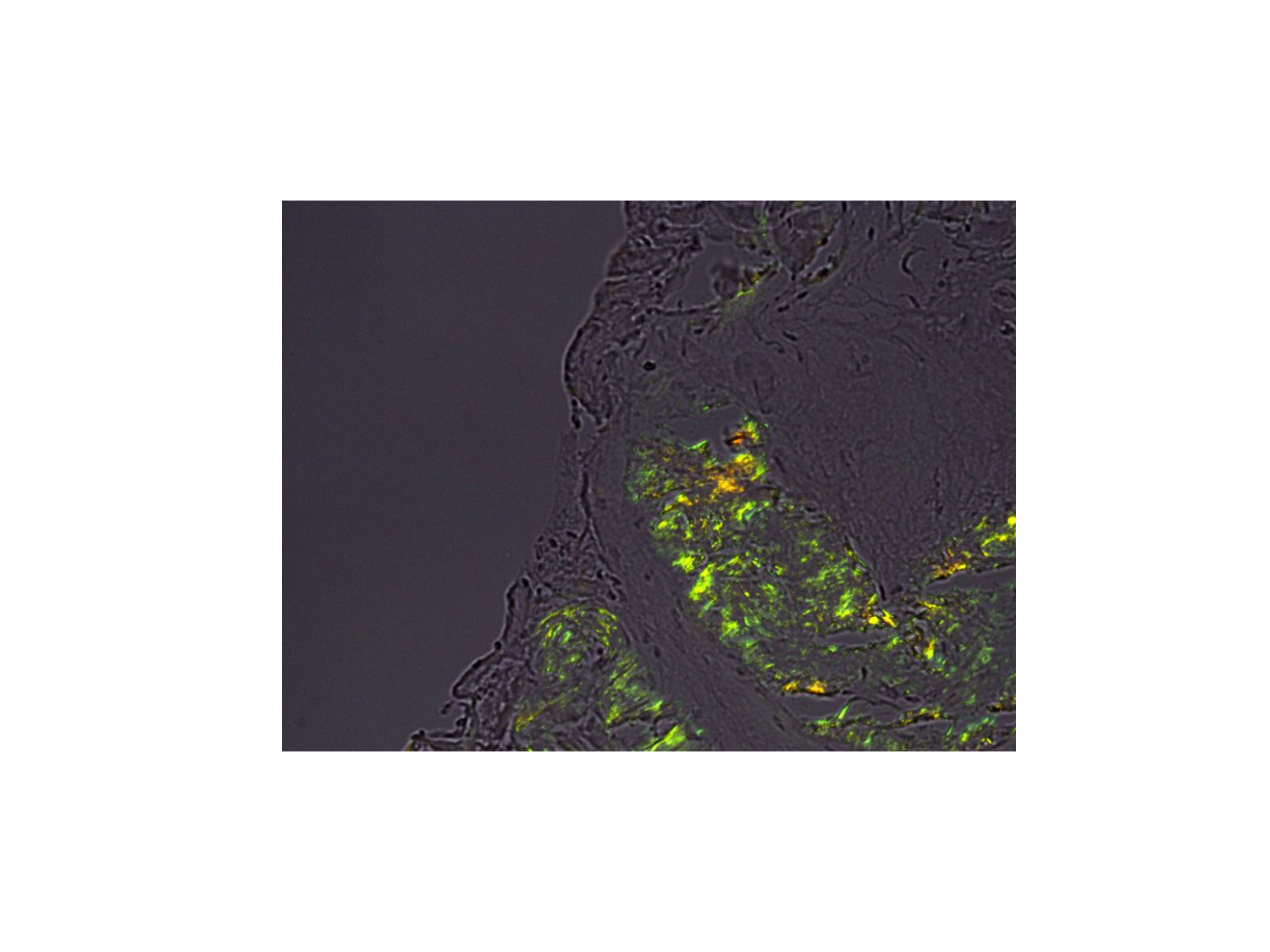

## Slide 5
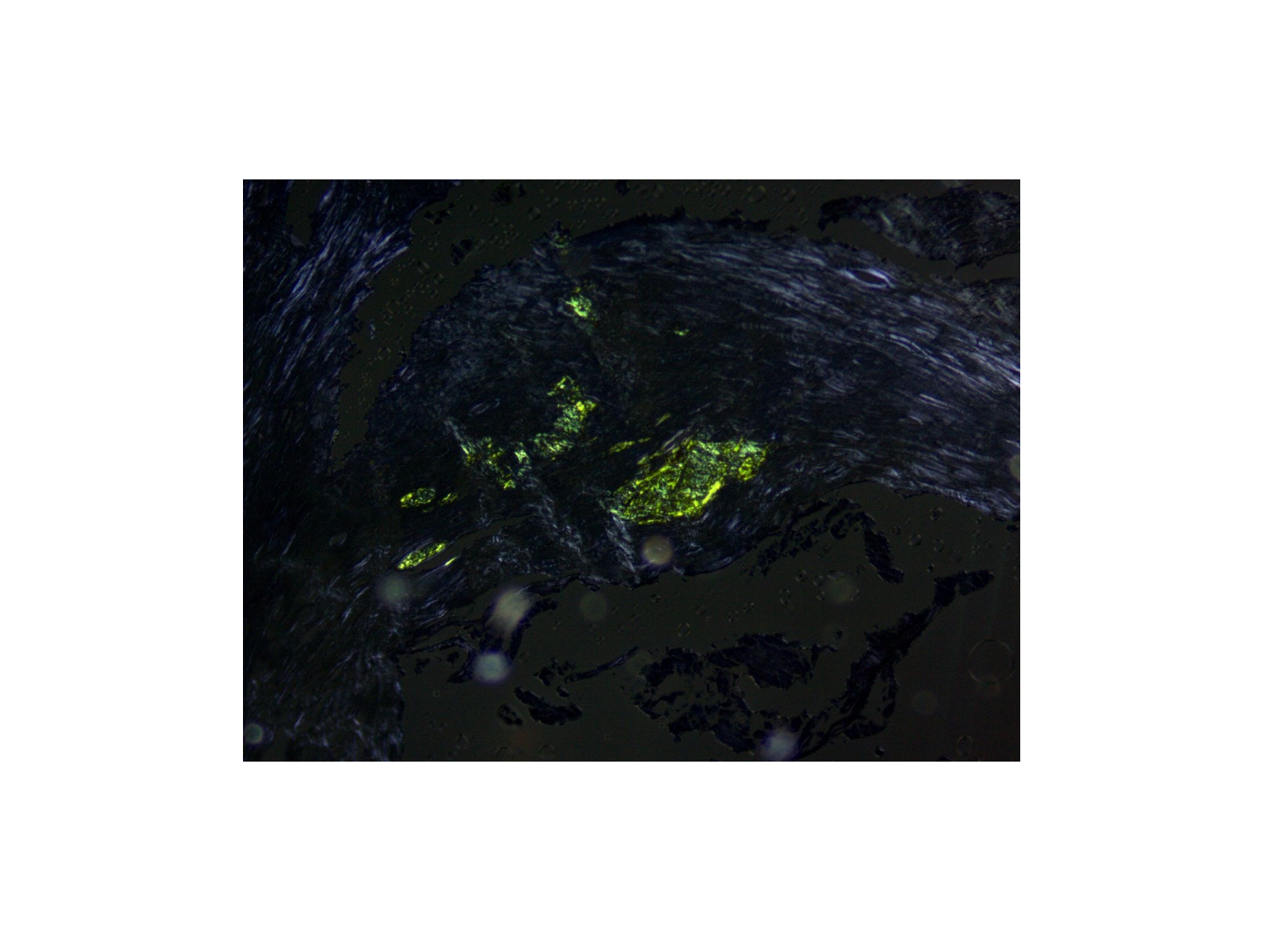

## Slide 6
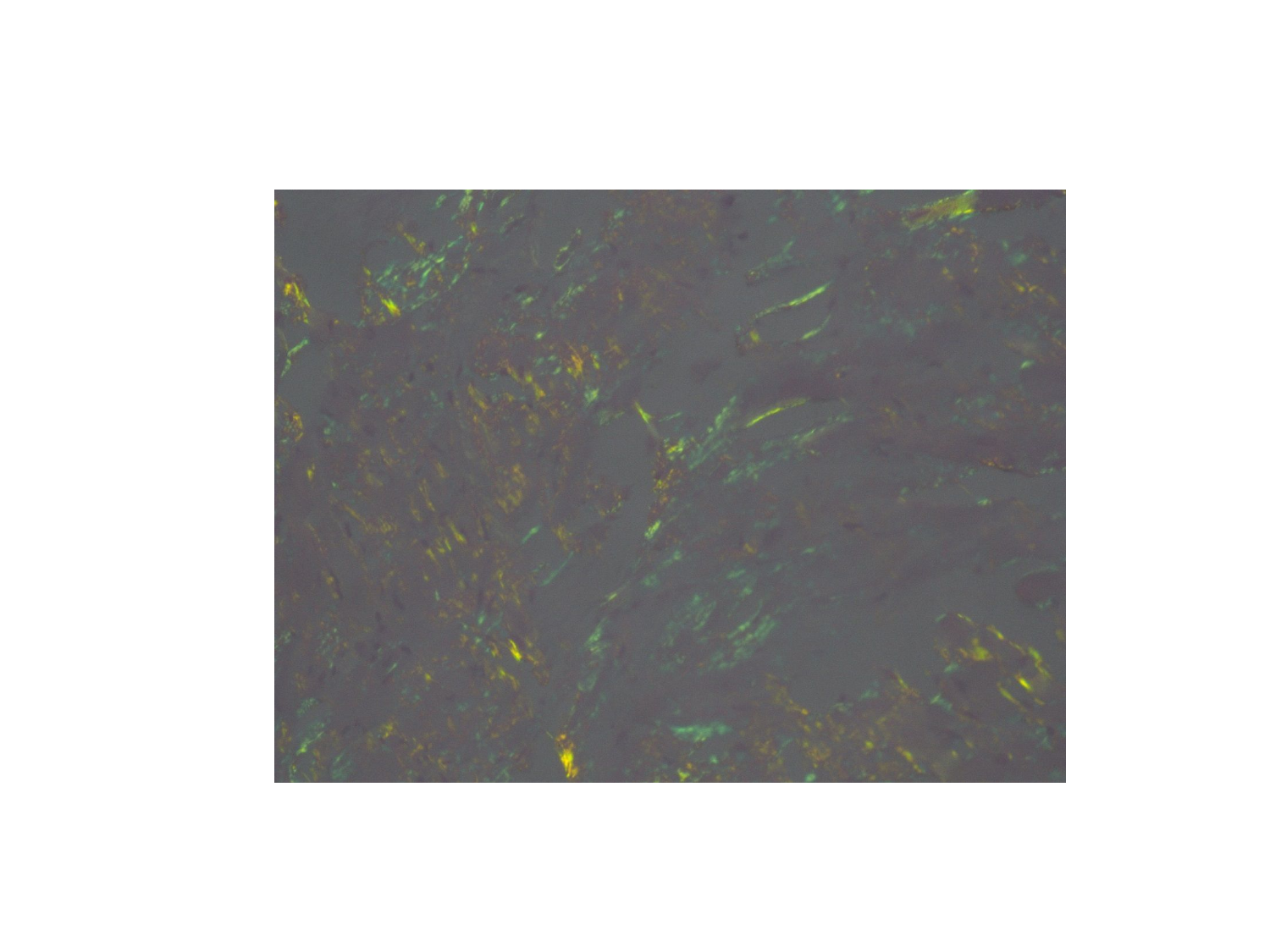

## Slide 7
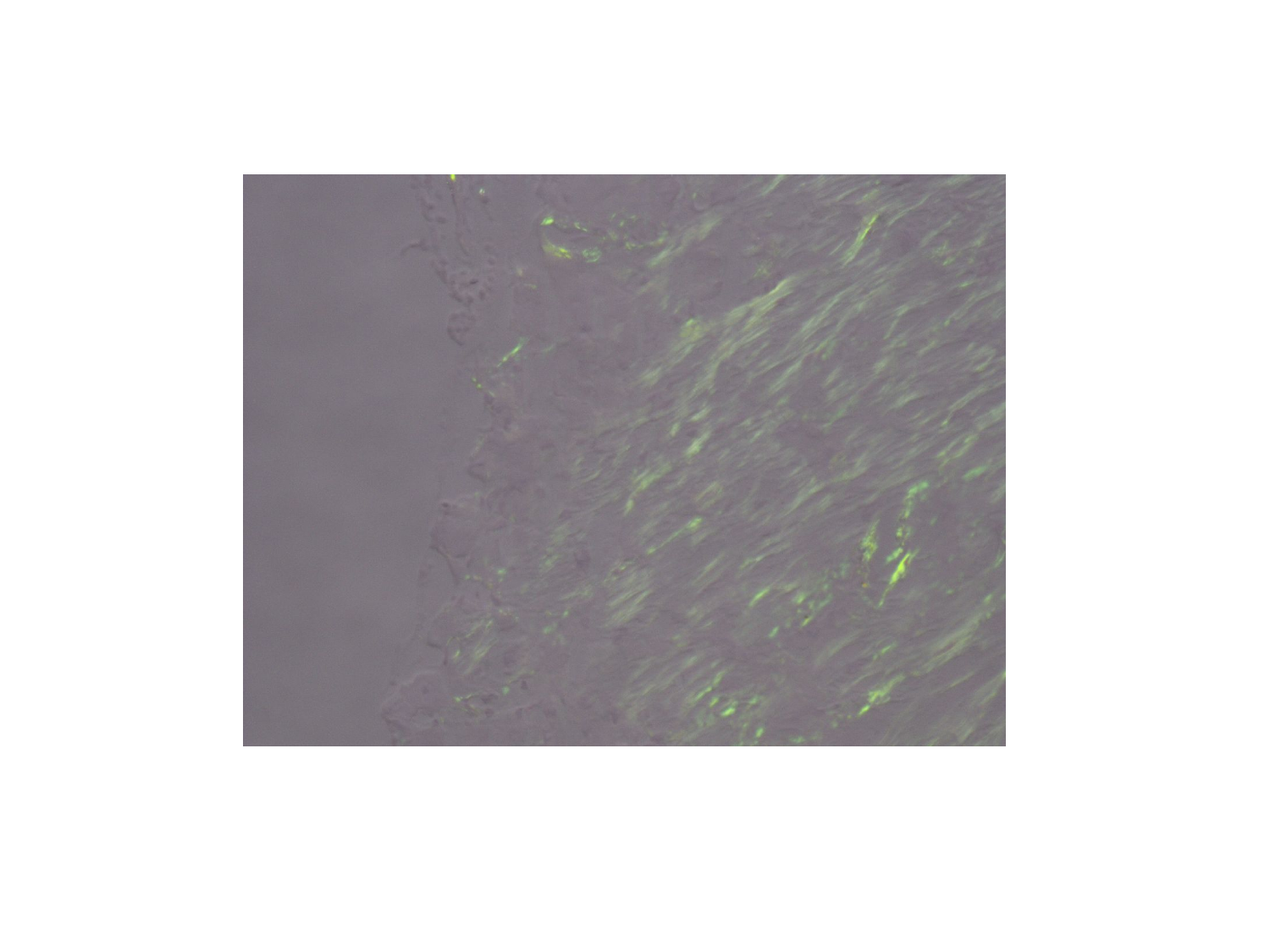

## Slide 8
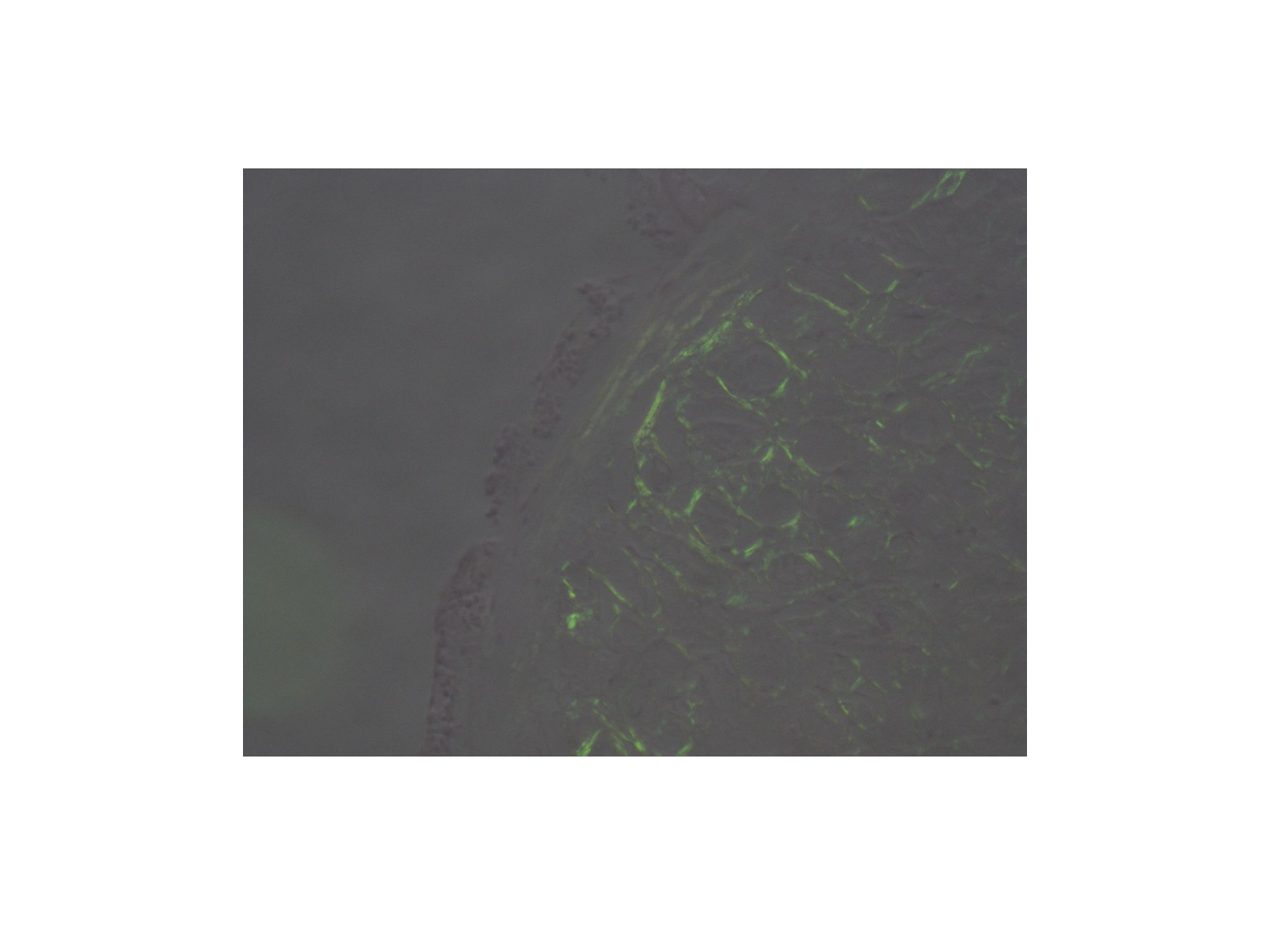

## Slide 9
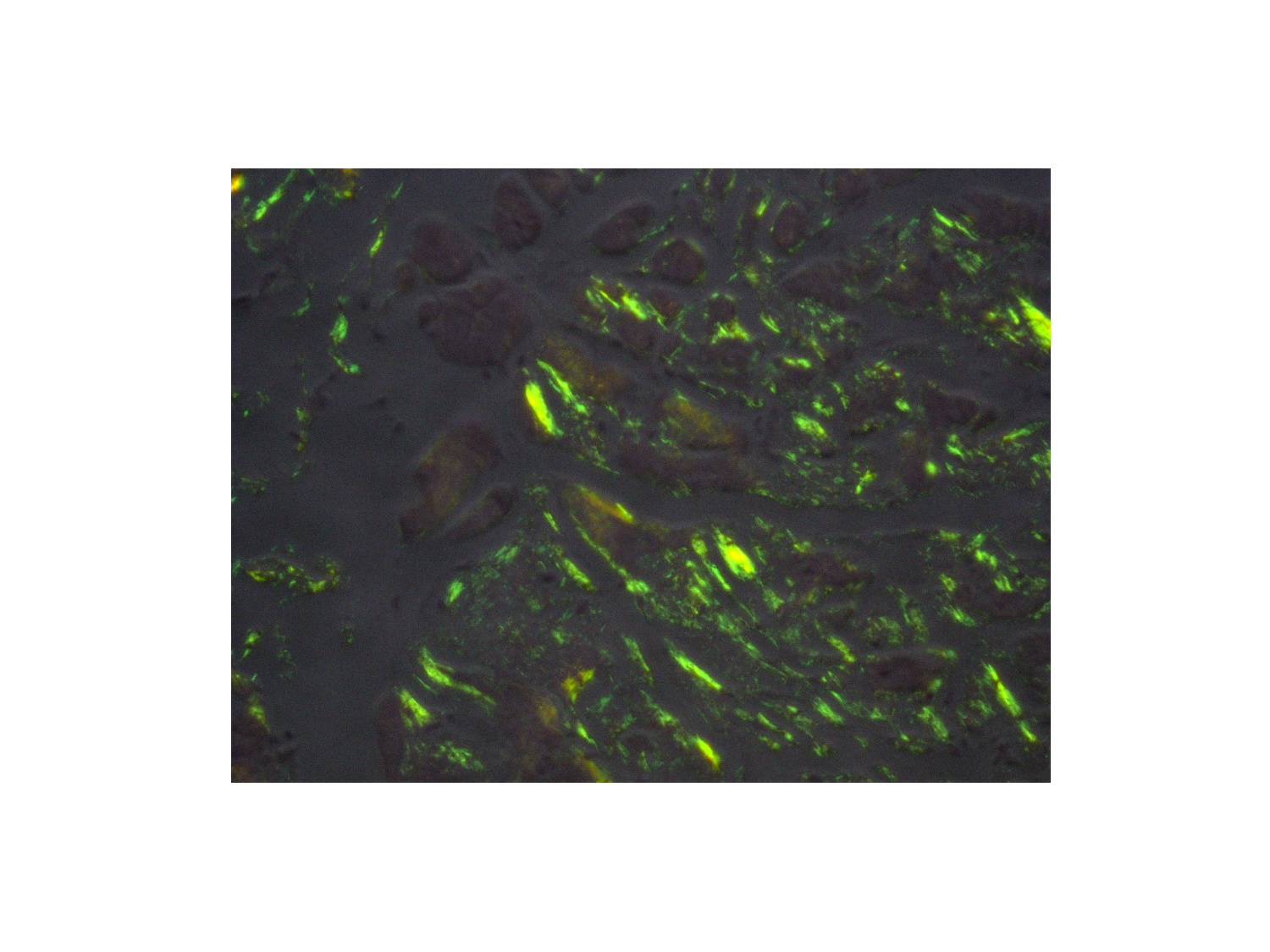

## Slide 10
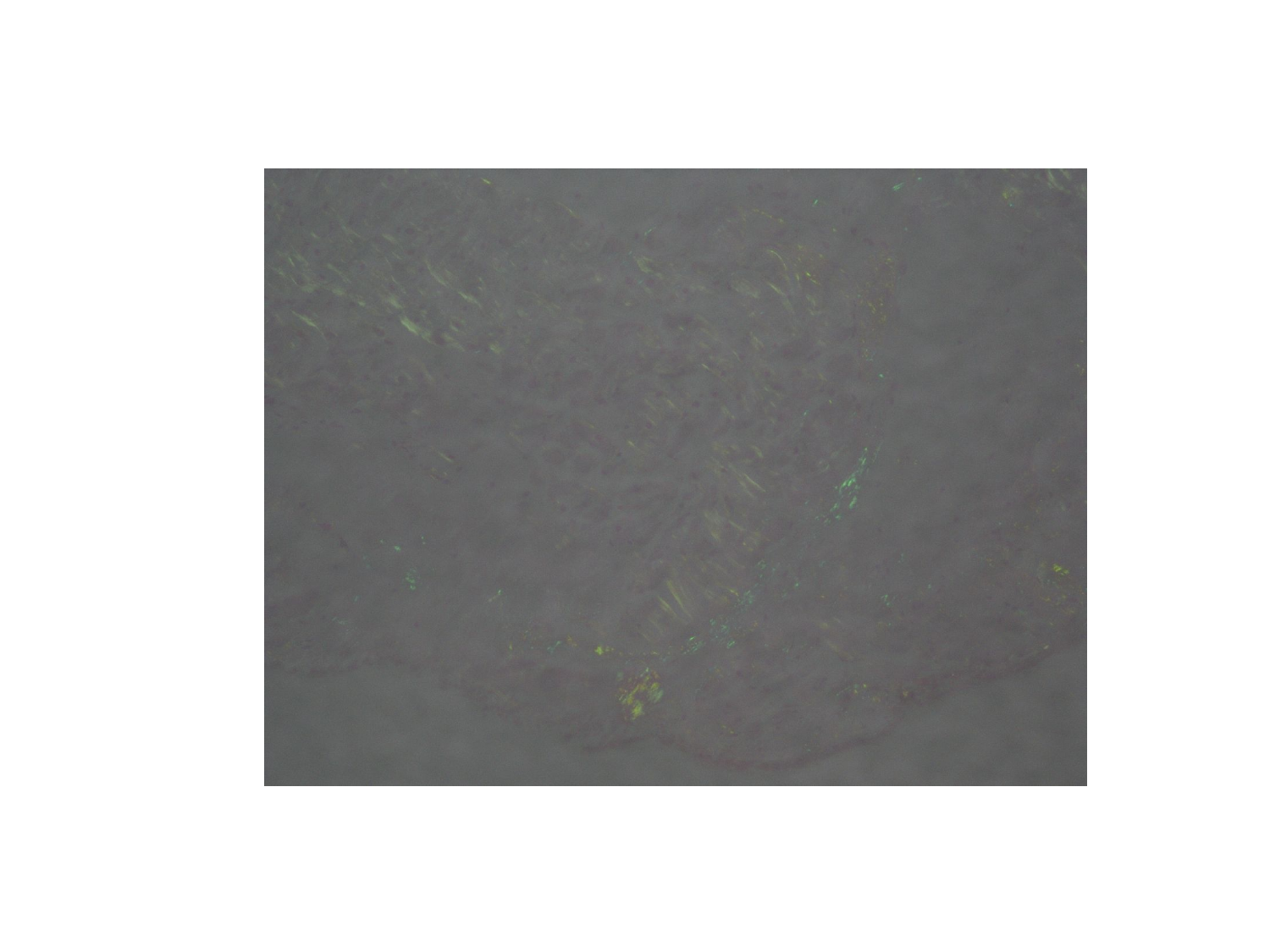

## Slide 11
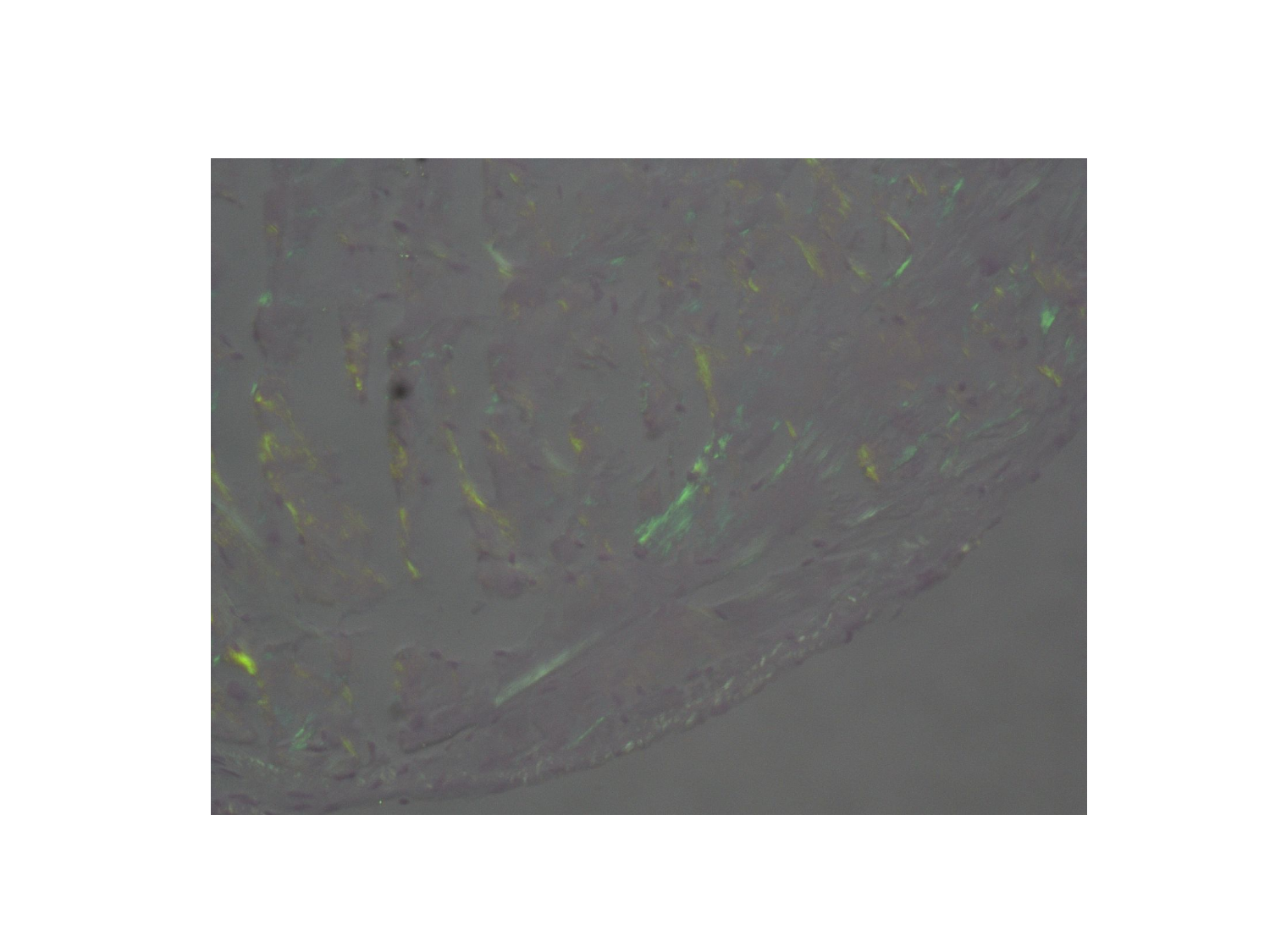

## Slide 12
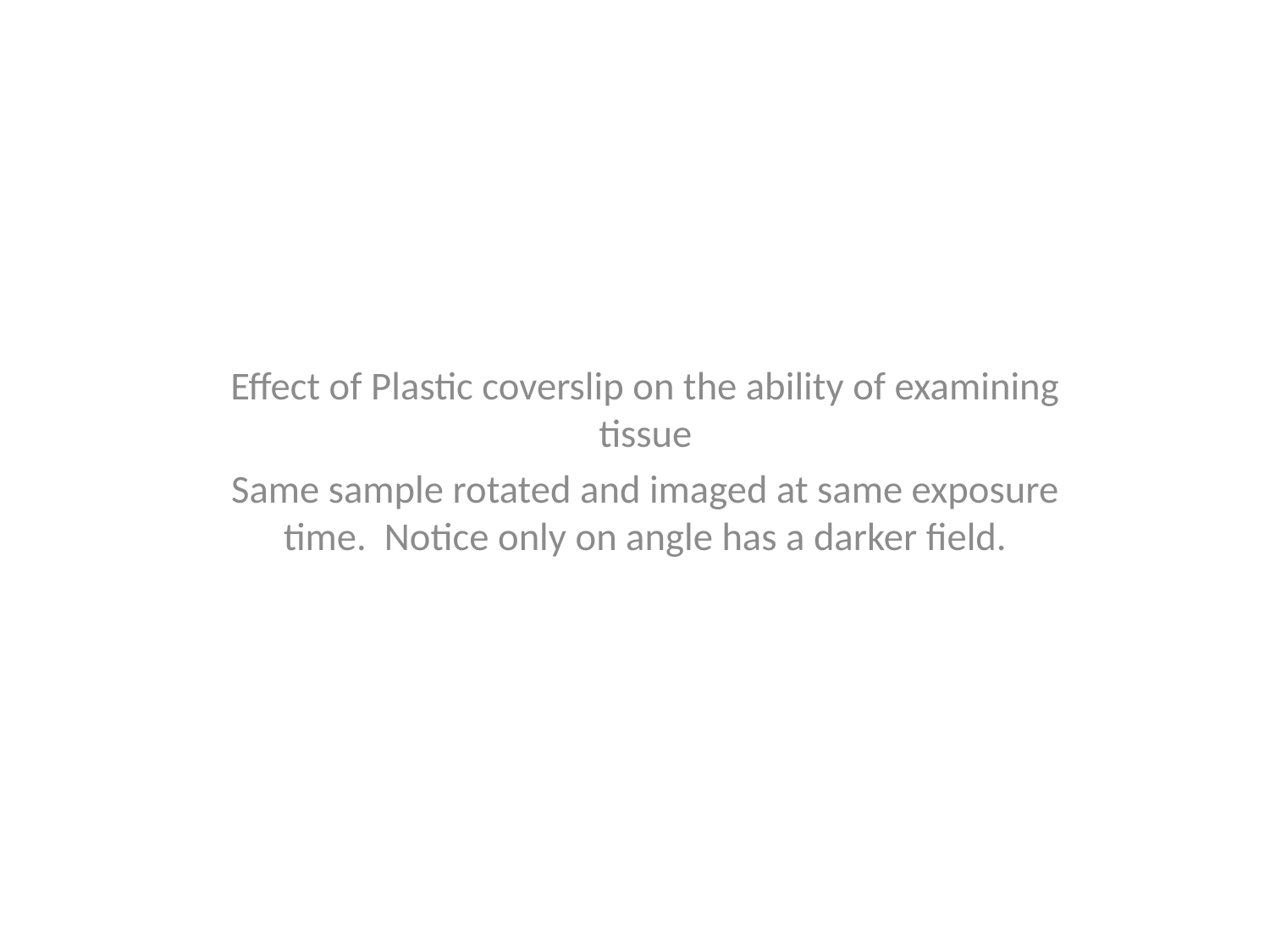

Effect of Plastic coverslip on the ability of examining tissue
Same sample rotated and imaged at same exposure time. Notice only on angle has a darker field.

## Slide 13
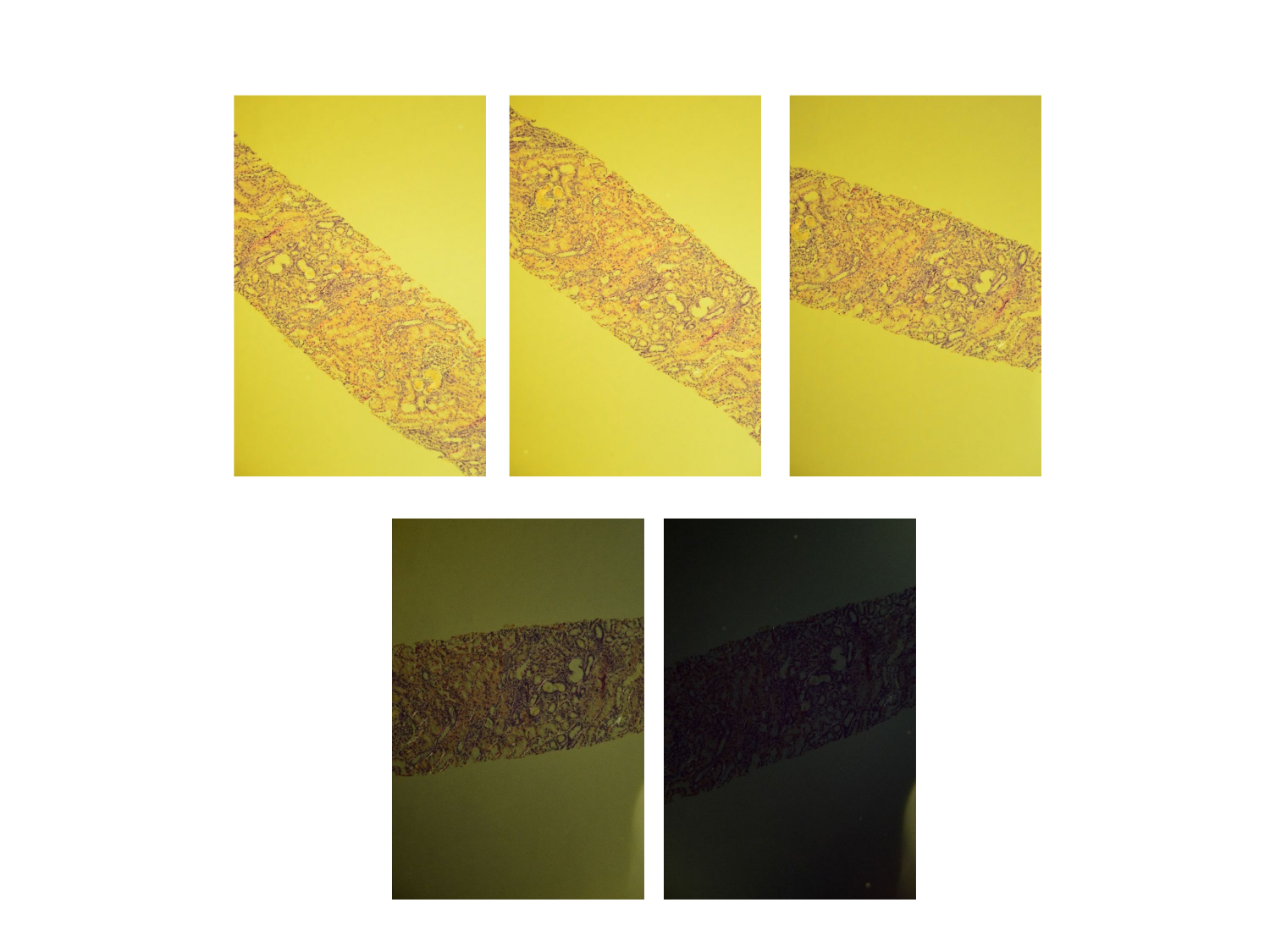

Supplement: Supplementary file 1 — Included a video recording of congored stained tissue covered by eith glass or plastic coverslips and rotated. (PPTX 11971 kb) [file 13000_2019_822_MOESM1_ESM.pptx]
